# Genome-Wide Investigation of Multifocal and Unifocal Prostate Cancer—Are They Genetically Different?

**DOI:** 10.3390/ijms140611816

**Published:** 2013-06-03

**Authors:** Chinyere Ibeawuchi, Hartmut Schmidt, Reinhard Voss, Ulf Titze, Mahmoud Abbas, Joerg Neumann, Elke Eltze, Agnes Marije Hoogland, Guido Jenster, Burkhard Brandt, Axel Semjonow

**Affiliations:** 1 Prostate Center, Department of Urology, University Hospital Muenster, Albert-Schweitzer-Campus 1, Gebaeude 1A, Muenster D-48149, Germany; E-Mail: Chinyere.Ibeawuchi@ukmuenster.de; 2 Center for Laboratory Medicine, University Hospital Muenster, Albert-Schweitzer-Campus 1, Gebaeude 1A, Muenster D-48149, Germany; E-Mail: Hartmut.Schmidt2@ukmuenster.de; 3 Interdisciplinary Center for Clinical Research, University of Muenster, Albert-Schweitzer-Campus 1, Gebaeude D3, Domagkstrasse 3, Muenster D-48149, Germany; E-Mail: voss@uni-muenster.de; 4 Gerhard-Domagk Institute of Pathology, University Hospital Muenster, Domagkstrasse 17, Muenster D-48149, Germany; E-Mail: Ulf.Titze@ukmuenster.de; 5 Institute of Pathology, University Hospital Hannover, Carl-Neuberg-Strasse 1, Hannover D-30625, Germany; E-Mail: Abbas.Mahmoud@mh-hannover.de; 6 Institute of Pathology, Klinikum Osnabrueck, Am Finkenhuegel 1, Osnabrueck D-49076, Germany; E-Mail: joerg.neumann@pathomail.de; 7 Institute of Pathology, Saarbrücken-Rastpfuhl, Rheinstrasse 2, Saarbrücken D-66113, Germany; E-Mail: e.eltze@pathologie-saarbruecken.de; 8 Department of Pathology, Erasmus Medical Center, ‘s-Gravendijkwal 230, 3015-CE Rotterdam, The Netherlands; E-Mail: a.m.hoogland@erasmusmc.nl; 9 Department of Urology, Erasmus Medical Center, ‘s-Gravendijkwal 230, 3015-CE Rotterdam, The Netherlands; E-Mail: g.jenster@erasmusmc.nl; 10 Institute for Clinical Chemistry, University Clinic Schleswig-Holsteins, Arnold-Heller-Strasse 3, Haus 17, Kiel D-24105, Germany; E-Mail: Burkhard.Brandt@uksh.de

**Keywords:** prostate cancer, multifocal, unifocal, copy number variation, heterogeneity, affymetrix

## Abstract

Prostate cancer is widely observed to be biologically heterogeneous. Its heterogeneity is manifested histologically as multifocal prostate cancer, which is observed more frequently than unifocal prostate cancer. The clinical and prognostic significance of either focal cancer type is not fully established. To investigate prostate cancer heterogeneity, the genetic profiles of multifocal and unifocal prostate cancers were compared. Here, we report observations deduced from tumor-tumor comparison of copy number alteration data of both focal categories. Forty-one fresh frozen prostate cancer foci from 14 multifocal prostate cancers and eight unifocal prostate cancers were subjected to copy number variation analysis with the Affymetrix SNP 6.0 microarray tool. With the investigated cases, tumors obtained from a single prostate exhibited different genetic profiles of variable degrees. Further comparison identified no distinct genetic pattern or signatures specific to multifocal or unifocal prostate cancer. Our findings suggest that samples obtained from multiple sites of a single unifocal prostate cancer show as much genetic heterogeneity and variability as separate tumors obtained from a single multifocal prostate cancer.

## 1. Introduction

Prostate cancer is the second most common cancer in men worldwide and the most frequently diagnosed cancer in men in Europe [1]. The complex and heterogeneous nature of prostate cancer remains a challenge that negatively influences clinical management of the disease. A major feature of prostate cancer complexity is the presence of multiple adenocarcinoma foci in 50% to 76% of radical prostatectomy specimens [2,3]. The multifocal status is identified during pathological evaluation as the presence of two or more cancer foci distinctly separated [2,3]. The unifocal prostate cancer has been described as a single anatomical cancer focus present in about 33% of radical prostatectomy specimens [4,5]. A report from Djavan *et al*. [5] associated high Gleason scores and tumor stage to multifocal prostate cancer and also proposed “multifocality” to be a predictor of recurrence [5]. In the view of many researchers, the multifocal nature of most prostate cancers has been blamed for the poorly predictable biological behavior of many cases and the difficulty in fully understanding its pathogenesis. [2,6,7].

In recent times, the use of microarray technologies has contributed immensely in prostate cancer research and generated a wealth of knowledge on the structural variations of the cancer genome. The dual-utilization of SNP (single nucleotide polymorphism) and CNV (copy number variation) markers in the Affymetrix SNP 6.0 microarray tool provides increased genomic resolution and an extensive mapping of the genome. This microarray tool is capable of identifying copy number changes and loss of heterozygosity, which have an influential role in altering gene expression levels, gene function and increasing disease susceptibility [8–10]. Exploiting the advantages of this high resolution SNP-CNV tool provides us with the possibility of investigating the prostate cancer genome and producing a characterized copy number profile for all tumors investigated.

With the growing need to understand the implication and existence of multifocal and unifocal prostate cancer, this study utilized radical prostatectomy specimens categorized by the number of tumor foci present. Forty-one fresh frozen prostate cancer samples and nine non-tumor samples from blood and normal prostate tissue were utilized. A high-resolution characterization of the tumor foci would enable the comparison of multifocal prostate cancers and unifocal prostate cancers to further investigate the heterogeneity of prostate cancer.

## 2. Results and Discussion

### 2.1. Chromosomal CNV Events

**Figure 1:**
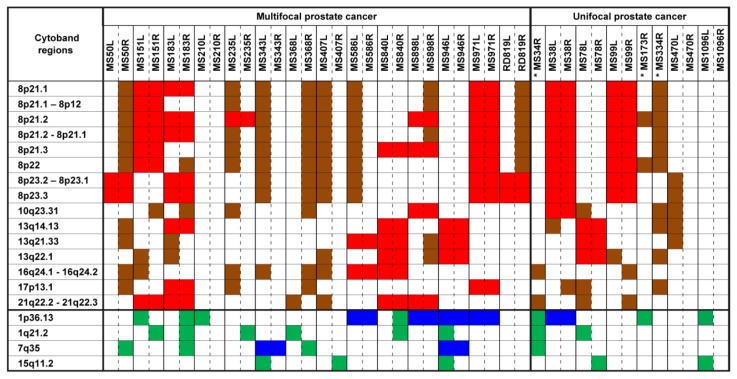
Most frequent copy number alterations in 41 tumor foci from 22 patients. (Color representations: red indicates losses observed in both tumor samples from a single prostate; brown indicates losses in one out of two tumor samples from a single prostate; blue indicates gains in both tumor samples from a single prostate; green indicates gains in one out of two tumor samples from a single prostate). (*) Matching left samples from these cases: MS34, MS173 and MS334 were not investigated, due to insufficient tumor quantity.

All tumor samples had a mean intensity QC (quality control) value of 96.5%, median of 96.79% and min-max range of 90.34%–98.94%. For the eight blood samples and one normal tissue sample, the mean QC value was 98.01%, median of 98.38% and min-max range of 96.79%–98.78%. The concordance check was conducted to ascertain that individual tumor pairs were indeed from the same prostate. All tumor pairs show concordance when called-SNPs were compared (Supplementary Information, Table S1). With the exclusion of CNV alterations present in both tumor and non-tumor samples, genome-wide CNV data obtained from 41 tumor samples showed somatic alterations (Figure 1) that were similar to earlier genomic studies [11–13]. Utilizing a minimum observation frequency of five tumors with CNV alterations, the most frequent gains were observed on these chromosomal regions: 1p36.13 (17/41, 41.5%), 1q21.2 (8/41, 19.5%), 7q35 (8/41, 19.5%) and 15q11.2 (5/41, 12.2%). Copy number losses were observed predominantly on chromosome regions 8p. The most recognized were 8p21.2 (22/41, 53.7%), 8p21.3 (20/41, 48.8%), 8p21.1 (19/41, 46.3%) and 8p21.2–8p21.1 (19/41, 46.3%). Other frequently observed losses were situated on 21q22.2–21q22.3 (13/41, 31.7%), 13q14.13 (12/41, 29.3%), 16q24.1–16q24.2 (12/41, 29.3%), 10q23.31 (11/41, 26.8%) and 17p13.1 (10/41, 24.4%) (Figure 1).

Chromosomal losses on 8p, 10q, 13q and 16q were observed most frequently in our study (Figure 1; Supplementary Information, Table S2). These aberrations correlate closely with CNV data from previous studies [14–16]. Notable chromosomal gains on 8q were not observed frequently in this study, but were identified in four distinct tumor foci specimens—MS50R, MS235L, MS368R and MS407L, which interestingly were tumors with high Gleason scores (Table 1). This observation does support the reported correlation between the gains observed on 8q and higher Gleason score [17,18]. In addition, low frequency of observed 8q gain may have been influenced by our tumor sample selection, which was restricted to organ-confined cancers, since 8q gains have been reported to be associated with advanced prostate cancer cases [17].

### 2.2. Comparison of Copy Number Profile Observed in Multifocal and Unifocal Prostate Cancers

**Figure 2:**
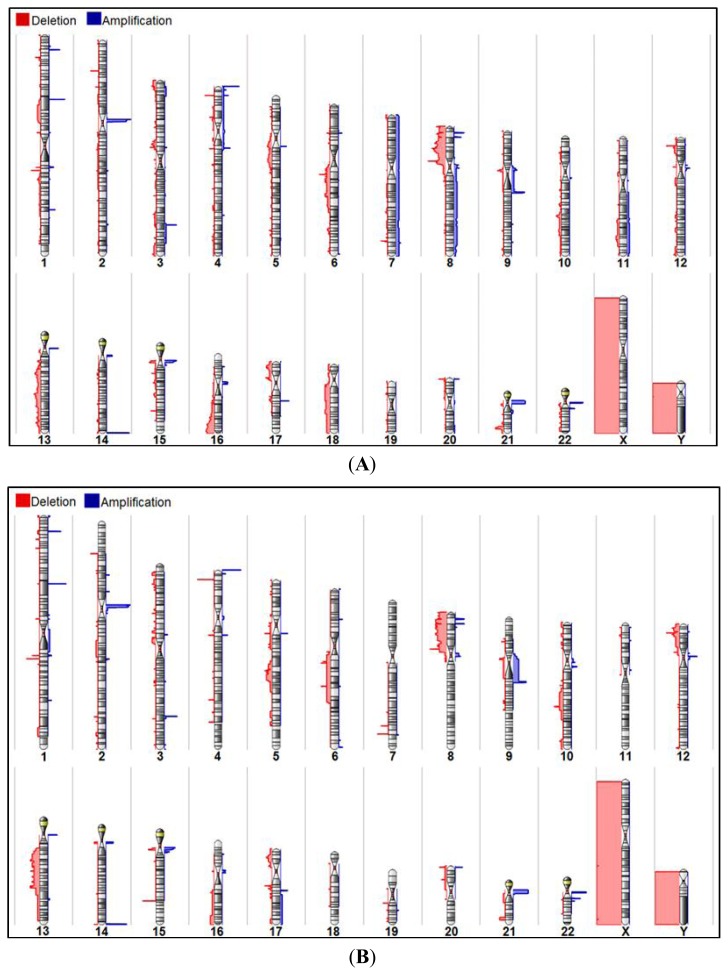
Summary of all tumors in a karyoview. (**A**) Multifocal prostate cancers; (**B**) Unifocal prostate cancers. Regions of blue and red are specific to regions of gains and losses, respectively.

Genome-wide analysis of 28 multifocal and 13 unifocal prostate cancer foci enabled the comparison of prostate cancer focal categories. To identify contrasting alterations, separate chromosomal karyoviews of multifocal and unifocal prostate cancers were scrutinized (Figure 2). Comparison identifies no exclusive chromosomal alteration that occurs frequently in any particular “focality” group. Frequent genetic alterations observed in multifocal prostate cancers were also present in some unifocal prostate cancers and *vice versa*. From the multifocal prostate cancer karyoview (Figure 2A), gains on chromosome 8q were observed in four tumor specimens (MS50R, MS235L, MS368R and MS407L). Though this aberration on 8q was not observed in any unifocal prostate cancer (Figure 2B), the low frequency of this event (*n* = 4) leaves it unspecific for multifocal prostate cancers specimens.

The absence of a clear-cut genetic difference between the multifocal and unifocal prostate cancers reaffirms that prostate cancer is a heterogeneous disease, and the genetic characteristics or biological behavior of a prostate cancer case is unlikely to be predicted by just the number of tumor foci present in the prostate. As already suggested by earlier studies, specific clonal cancer cell populations may be responsible for the aggressiveness of some prostate cancer cases [2,19]. Our data support these assumptions, and we also suggest that aggressive potential can be found in both multifocal and unifocal prostate cancers and cannot be specifically attributed to a distinct “focality” group.

### 2.3. Hierarchical Clustering

**Figure 3:**
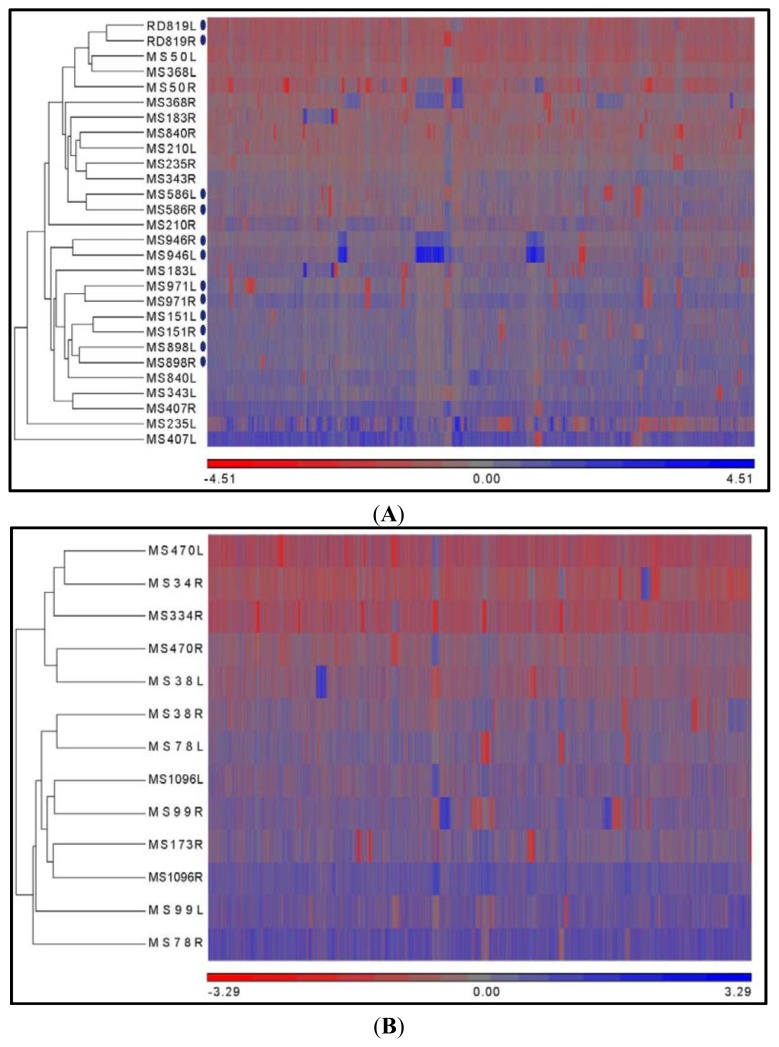
Hierarchical clustering of copy number summary from chromosome 1 to 22. (Heat map: red represents gene losses, blue represents gene gains and grey represents unchanged gene states). Blue oval symbols denote tumors from the same prostate that remain closely clustered. (**A**) Multifocal prostate cancers; (**B**) Unifocal prostate cancers.

The copy number variation data of samples from multifocal and unifocal prostate cancer were subjected to hierarchical clustering (Figure 3). Copy number losses and gains located on chromosomes 1 to 22 were utilized in the Partek^®^ Genomic Suite environment (St. Louis, MO, USA). Due to artificial copy number loss on the X and Y chromosome, the genetic data on these chromosomes were not utilized for the clustering process. The hierarchical clustering of all cases shows clusters of tumors with congruent copy number profiles.

**Figure 4:**
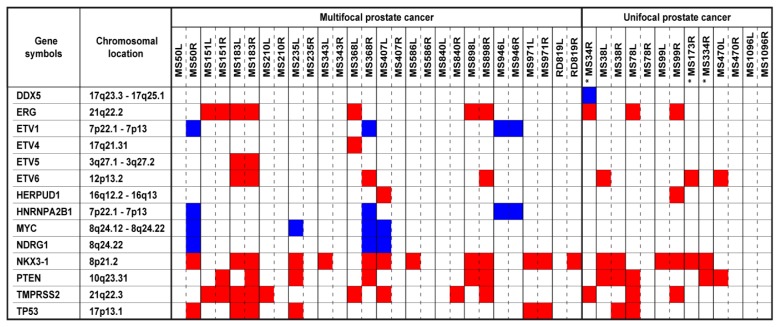
Observed copy number altered genes in investigated tumors. Red represents losses and blue represents gains. Gene list obtained from http://www.sanger.ac.uk/genetics/CGP/Census/[22]. (*) Matching left samples from these cases—MS34, MS173 and MS334—were not investigated, due to insufficient tumor quantity.

Six tumor pairs from these multifocal prostate cancers—MS151, MS586, MS898, MS946, MS971 and RD819—were closely clustered, due to strong similarities in CNV profiles (Figure 3A). These tumor pairs may have progressed from a single cancer cell population, because of their largely similar genetic profiles. However, some potentially critical genetic differences were detected and highlighted in Figure 4. The loss of PTEN was found distinctly in the right tumor of MS151, the loss of NKX3 -1 was located in the left tumor of MS586 and the right tumor of RD819 and the loss of ETV6 and TMPRSS2 was found in the right tumor of MS898. These observations show that some tumor pairs from single prostates share several genetic similarities, but still possess few and possibly critical genetic differences. With the other cases, multifocal tumors from different patients were observed to be closely clustered. Here, tumor pairs from the same prostate were not found on the same cluster, due to dissimilar CNV profiles. Multifocal tumor pairs with the most dissimilar CNV profiles (MS50, MS235, MS343, MS368 and MS407) indicated an evolution of tumor foci from separate clonal cell ancestry. The presence of separate tumor foci has been described to originate from different cell origins, advance at dissimilar rates and, thus, accumulating very different degrees of genetic alterations [6,20]. With these observations, it is revealing that within multifocal prostate cancer, tumor foci can be variably different; while some tumor foci from a single prostate share large genetic alteration, other tumor foci have very few genetic alterations in common.

The unifocal prostate cancers possess larger tumor volumes than the individual tumor foci in multifocal prostate cancers (Table 1). The unifocal tumors have a mean tumor volume of 13.6 cm^3^, while the multifocal tumor foci have a mean tumor volume of 2.5 cm^3^. Analyzed tumors obtained from two flanks of a single focus showed that tumor pairs from these unifocal prostate cancer specimens—MS38, MS78, MS99, MS470 and MS1096 (Figure 3B)—possess dissimilar genetic profiles, *i.e*., tumor pairs from a single focus were not present in a single cluster. From the cluster diagram (Figure 3B), the unifocal prostate cancer specimens exhibited heterogeneity within the single focus, and it has been suggested to be borne from the possible confluence of multiple separate tumor foci. Unifocal prostate cancer can be said to be initiated by the progression of independent smaller tumor foci, from which, as time elapses, the tumor foci merge to form a tumor mass, which has a much larger tumor volume and possesses different genetic alterations depending on the area of the tumor investigated [6,7]. It was also suggested that unifocal prostate cancer may be a phenotypic representation of prostate cancer in its late stages [21]. In this study, unifocal prostate cancer was observed to be as heterogeneous as multifocal prostate cancer, due to the possible fusion of several tumor foci of possibly different biological characteristics.

### 2.4. Copy Number Altered Genes Elucidate Prostate Cancer Heterogeneity

Our study also reveals that alteration of some notable genes was not observed uniformly in tumor pairs from multifocal and unifocal prostate cancer specimens. Genes selected from the Cancer Genome Project [22] and reported to be frequently altered in prostate cancer [23] were detected variably in tumor pairs from single prostates (Figure 4). The presence of “altered prostate cancer -related genes” elucidates the genetic heterogeneity in multifocal and unifocal prostate cancers.

## 3. Experimental Section

### 3.1. Sample Acquisition, Handling and Preparation

Fresh frozen radical prostatectomy specimens from over 1700 prostate cancer patients were archived between the years 1998 to 2007 in the bio-bank of the Department of Urology, University Hospital, Muenster, with informed consent of the patients. Prior to storage of tissue specimens, prostate tissue portions from the left and right apex region were embedded in “Tissue -Tek” OCT (optimum cutting temperature) compound (Sakura Finetek, Torrance, CA, USA), briefly flash-frozen in liquid nitrogen and stored afterwards in 2-Methylbuthane solution at −80 ºC. Preserving these tissue portions from the apex region was based on evidence that tumors occur frequently in the peripheral zone and extend laterally into the apex region [24].

**Figure 5:**
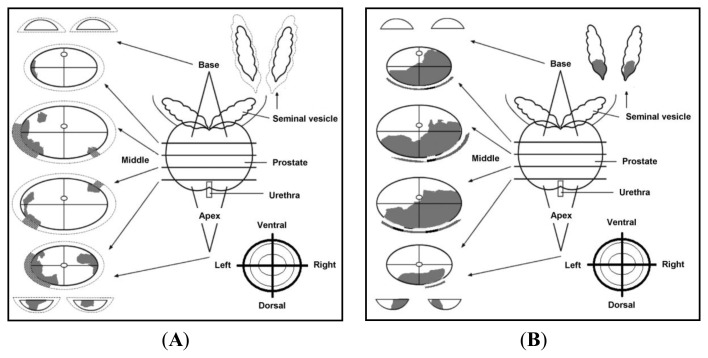
Institutional routine pathology report. The documents contain cross-sectional views of radical prostatectomy specimens showing areas of adenocarcinoma in the prostate pathological mapping of (**A**) a multifocal prostate cancer and (**B**) a unifocal prostate cancer [24].

All deposited specimens in the Department of Urology, University Hospital, Muenster, were accompanied with pathological reports, which contained cross-sectional views of whole prostates and mapped areas of adenocarcinoma (Figure 5). With the advantage of illustrative pathological reports, the major criterion for case selection was the presence of tumor in the apex region of the prostate, where the fresh frozen samples were taken. Tumor foci separated by benign tissue in the apex region were necessary for selection of multifocal prostate cancer cases. Unifocal prostate cancers should possess a single tumor focus that extended into the apex region. Specimens obtained from the Erasmus Medical Center, Rotterdam, were selected based on written pathological reports, which held information on the anatomical location of tumor(s). From all deposited tissue specimens, 221 cases were selected, cut and pathologically evaluated by experienced pathologists (Elke Eltze; Mahmoud Abbas; Joerg Neumann; Ulf Titze and Geert J.L.H. van Leenders).

All tissue samples and section preparations were conducted at −20 °C in a MTC bench-top cryocut machine (SLEE, Mainz, Germany). Tissue sections of 5 μm bordering the area of interest were cut and stained with hematoxylin and eosin (H&E). Stained tissue sections were afterwards reviewed by the pathologists. Areas of adenocarcinoma were marked on the slide(s), and unwanted tissue portions were macro-dissected. Repetition of the procedure was carried out until the tumor tissues reached a maximum achievable percentage of approximately 80%–100%.

From the initial selection of 221 cases, prostate cancer specimens from 22 patients were afterwards selected based on sufficiently high tumor quantity and utilized in this study. Also included in this collective are additional samples obtained from a single prostate cancer patient treated at the Erasmus Medical Centre (EMC), Rotterdam (Table 1). Forty-one individual tumor specimens were obtained from these 22 patients. Twenty-eight tumor foci were obtained from 14 multifocal prostate cancers, and 13 tumor specimens were obtained from eight unifocal prostate cancers. Multifocal prostate cancers refer to patients’ specimen with two or more adenocarcinoma foci, separated by normal tissue, while unifocal prostate cancer consisted of a single tumor focus. In the multifocal prostate cancer collective, distinct tumors were obtained from the left and right part of the prostate’s apex region. With the unifocal prostate cancer collective, the tumor specimens were excised from the left and right side of the single tumor focus. Furthermore, nine non-tumor specimens were utilized in this study, which were comprised of eight blood samples and one histological benign tissue (Table 1).

From Table 1, it can be observed that investigated tumors were obtained from specimens with rather high tumor volume. The utilization of these cases may imply a selection bias; however, these specimens were necessary in order to obtain sufficient tumor percentage needed for experiments. Furthermore, it is important to emphasize that many multifocal tumors were not present with two separate foci in the apex region, *i.e*., more than 1,700 radical prostatectomy specimens had to be screened to find the relatively small number of samples that was investigated in this study.

### 3.2. DNA Preparation and Quality Control

Genomic tumor and blood cell DNA were isolated using standard protocols from the Maxwell^®^ Promega 16 reagent kits and Maxwell^®^ Promega personal automated system (www.promega.com; Mannheim, Germany) [25], which ensured no cross-contamination. DNA obtained was measured for quality by spectrophotometric absorbance at 260 nm and 280 nm and by 1% agarose gel electrophoresis to assess high DNA molecularity. The microarray chips and reagents were purchased from Affymetrix (Santa Clara, CA, USA) and other manufacturers recommended by Affymetrix (Santa Clara, CA, USA). The experiments were conducted according to the Affymetrix Genome-Wide^®^ Human SNP Array 6.0 standard protocol (Santa Clara, CA, USA).

### 3.3. Genome-Wide Microarray Analysis

Two-hundred fifty nanograms/five microliters of genomic DNA were digested with Nsp and Sty restriction enzymes (New England Biolabs, Herts, UK), ligated with Nsp and Sty adaptors (Affymetrix, Santa Clara, CA, USA) and amplified in the PCR step. PCR products of 250–1000 base pair (bp) fragments were purified, fragmented to lengths of 50–200 bps and labeled with biotinylated dideoxy ATP (Affymetrix, Santa Clara, CA, USA). These labeled probes were hybridized to the Affymetrix 6.0 SNP-CNV microarray chip (Santa Clara, CA, USA) at 50 °C, 60 rpm for 16 to 18 h. These were subsequently washed, stained and scanned with the Affymetrix Genechip^®^ Scanner 3000 (Santa Clara, CA, USA). With the Affymetrix Genotyping Console (version 4.1.2) software [26], each chip experiment was quality controlled for contrast and intensity. Data produced as CEL files were exported for further analysis with the Partek^®^ Genomic Suite software (version 6.6) (Partek Incorporated, St. Louis, MO, USA) [14,27].

### 3.4. Data Analysis

Using the Partek Genomic Suite (St. Louis, MO, USA), all experimental CEL files were normalized against a universal reference (Hapmap 270 control samples) to analyze chromosomal regions gained and lost. Present on the Affymetrix microarray chip are CNV and SNP probes. For a wider and higher resolution of the genome, the intensity of both SNP and CNV probes were interrogated for copy number analysis by the genomic segmentation algorithm. The parameters for the execution of the genomic segmentation algorithm include: minimum genomic markers/probes of 100, *p*-value of ≤0.001 and signal-to-noise ratio of 2 ± 0.3 (limits of detecting the normal range in a diploid region: 1.7 to 2.3). The Genomic segmentation algorithm [14,28] conducts its task of copy number analysis in two steps: firstly, a breakpoint was established, and secondly, the aberration status of that chromosomal region was ascertained. A breakpoint is recognized when a two-sided *t*-test statistically compares two neighboring regions/segments and there is a significant change in chromosomal abundance (*p* < 0.001). The aberration status of the region is thus established when a one-sided *t-*test was used to statistically compare the probe distributions mean of the chromosomal regions and the expected range of the normal (1.7 to 2.3). Annotations of significant regions were conducted with Refseq [29,30], and subsequent data handling was conducted with Microsoft^®^ Office Excel 2010 (Microsoft Corporation, WA, USA). Hierarchical clustering was performed with the Euclidean [31] distance measure, which determines sample dissimilarity and average linkage.

## 4. Conclusions

Our current results show that no genetic characteristics were identified that differentiate between multifocal and unifocal prostate cancers. Further observations show that prostate cancer is a heterogeneous disease. The variations in genetic alterations observed in a single prostate draw attention to the multi-faceted and complex events that occur in prostate carcinogenesis. We propose that the problem of heterogeneity is not specific to multifocal prostate cancers, but to unifocal prostate cancers, as well. The genetic inconsistencies within a tumor focus and among tumor foci pose a challenge to comprehensive knowledge of prostate cancer biology, clinical management of prostate cancer and future research studies.

## Supplementary Information



## Figures and Tables

**Table 1:** Clinical and pathological information of investigated tumors.

Sample ID	Patient age at time of surgery (years)	Total Gleason score	Gleason score of individual focus	Pathological stage	Clinical stage	Total tumor volume (cm^3^)	Tumor volume of individual focus (cm^3^)	Focality	Number of tumor foci in prostate
MS50L	66	4 + 3	3 + 4	pT3a	cT2c	2.1	0.7	multifocal	2
MS50R			4 + 3				1.4		

MS151L	61	4 + 3	4 + 3/3 + 3	pT3b	cT2c	2.85	0.76	multifocal	2
MS151R			4 + 3				2.09		

MS183L	59	4 + 3	3 + 4	pT3a	cT2b	7.35	0.35	multifocal	2
MS183R			3 + 4				7.0		

MS210L	62	3 + 3	3 + 3	pT3b	cT2c	8.64	4.16	multifocal	2
MS210R			3 + 3				4.48		

MS235L	65	4 + 5	4 + 4/4 + 5	pT3a	cT2b	12.5	12.0	multifocal	2
MS235R			3 + 3/3 + 4				0.5		

MS343L	57	2 + 3	3 + 3	pT2b	cT2b	1.33	0.95	multifocal	3
MS343R			3 + 4/3 + 3				0.19		

MS368L	65	4 + 5	3 + 4	pT3c	cT2b	13.76	2.56	multifocal	2
MS368R			4 + 5				11.2		

MS407L	51	3 + 4	3 + 4/4 + 3	pT2b	cT2c	1.35	0.81	multifocal	2
MS407R			3 + 3/3 + 4				0.54		

MS586L	53	3 + 2	3 + 3	pT2c	cT1c	4.55	0.7	multifocal	5
MS586R			3 + 3				1.4		

MS840L	66	4 + 3	3 + 4	pT3a	cT2c	2.64	1.44	multifocal	4
MS840R			3 + 4				0.48		

MS898L	54	4 + 3	3 + 4/3 + 3	pT2c	cT1c	3.5	0.35	multifocal	3
MS898R			3 + 4				2.8		

MS946L	61	3 + 2	3 + 3	pT3a	cT2b	6.75	6.3	multifocal	2
MS946R			3 + 3				0.45		

MS971L	50	4 + 5	3 + 4	pT3a	cT2a	1.26	1.08	multifocal	2
MS971R			3 + 4				0.18		

RD819L	52	NA	3 + 3	pT3a	cT1c	NA	NA	multifocal	2
RD819R			3 + 3				NA		

*MS34L	71	4 + 3	NA	pT2a	cT1c	4.56		unifocal	1
MS34R			4 + 3						

MS38L	62	3 + 4	3 + 3	pT3c	cT2c	22.4		unifocal	1
MS38R			3 + 3						

MS78L	66	3 + 3	3 + 3	pT3a	cT2b	3.08		unifocal	1
MS78R			3 + 3						

MS99L	57	3 + 4	3 + 3	pT3a	cT2c	4.42		unifocal	1
MS99R			3 + 3						

*MS173L	59	3 + 3	NA	pT3b	cT2b	1.2		unifocal	1
MS173R			3 + 3						

*MS334L	67	4 + 4	NA	pT3c	cT2c	15.96		unifocal	1
MS334R			3 + 3						
MS470L	64	3 + 4	3 + 3	pT3a	cT2b	11.27		unifocal	1
MS470R			3 + 3						
MS1096L	63	5+4	3+4/4+4	pT4	NA	45.75		unifocal	1
MS1096R			3+3						

Sample ID: MS = Muenster Bio-bank, RD = Rotterdam Bio-bank, location of prostate from which the tumor foci was obtained (L = left, R = right). Where two Gleason scores (GS) are stated: GS of upper cut-section of tumor/GS of bottom cut-section of tumor. Matching blood specimens were analyzed from MS50, MS151, MS368, MS840, MS971, MS34, MS334, MS38 and normal prostate tissue from RD819.

* MS34L, MS173L and MS334L had insufficient tumor quantity and were thus excluded. Full table: Supplementary Information, Table S3.
