# Supplementary material for: Genome-Wide Investigation of Multifocal and Unifocal Prostate Cancer—Are They Genetically Different?
Source: Int J Mol Sci. 2013 Jun 3;14(6):11816–29. doi: 10.3390/ijms140611816 (PMC3709757; doi:10.3390/ijms140611816)
Supplement: Supplementary file 1 [file ijms-14-11816-s001.pdf]

Supplementary Information

Table S1. Concordance between tumors obtained from the same prostate. With the Affymetrix Genotyping console, samples obtained from left and right parts of multifocal and unifocal prostate cancers were analyzed for concordance by utilizing called SNPs (single nucleotide polymorphisms). Percent of concordance above 95% indicates that tumor pairs are concordant and are from the same patient.

Focal groups	Tumor 1	Tumor 2	# SNPs Called	# Concordant SNPs	Concordance(%)
Multifocal prostate cancers	MS50L	MS50R	889367	887885	99.83
	MS151L	MS151R	880129	877810	99.74
	MS183L	MS183R	878329	871493	99.22
	MS210L	MS210R	873087	861764	98.7
	MS235L	MS235R	870383	857435	98.51
	MS343L	MS343R	868215	858846	98.92
	MS368L	MS368R	891865	889358	99.72
	MS407L	MS407R	850248	835899	98.31
	MS586L	MS586R	883709	880514	99.64
	MS840L	MS840R	875854	866243	98.9
	MS898L	MS898R	875292	871653	99.58
	MS946L	MS946R	873157	866811	99.27
	MS971L	MS971R	869438	863180	99.28
	RD819L	RD819R	890003	888132	99.79
Unifocal prostate cancers	MS38L	MS38R	880539	875184	99.39
	MS78L	MS78R	870073	862441	99.12
	MS99L	MS99R	863543	850650	98.51
	MS470L	MS470R	888624	884144	99.5
	MS1096L	MS1096R	867767	861224	99.25

Table S2. Tumor-specific copy number gains and losses. Cytoband regions with copy number alterations and annotated genes. CNV, copy number variation.

Cytoband region	CNV event	Number of tumors (*n* = 41)	Genes annotated to tumor-specific altered cytoband region
**1p36.13**	gain	17	*NBPF1*
**1q21.2**	gain	8	*NBPF14, NBPF15, NBPF16, PPIAL4A, PPIAL4B, PPIAL4C, PPIAL4D, PPIAL4E, PPIAL4F*
**7q35**	gain	8	*ARHGEF35, ARHGEF5, CTAGE4, FAM115A, FAM115C, NOBOX, OR2A1, OR2A12, OR2A14, OR2A2, OR2A25, OR2A42, OR2A5, OR2A7, OR2F1, OR2F2, OR6B1, TPK1*
**15q11.2**	gain	5	*HBII-52-27, HBII-52-28*
**8p21.2**	loss	22	*ADAM28, ADAM7, ADAMDEC1, ADRA1A, BNIP3L, CDCA2, DOCK5, DPYSL2, EBF2, GNRH1, KCTD9, NEFL, NEFM, NKX2-6, NKX3-1, PNMA2, PPP2R2A, STC1*
**8p21.3**	loss	20	*ATP6V1B2, BMP1, CSGALNACT1, DOK2, EPB49, FAM160B2, FGF17, GFRA2, HR, INTS10, LGI3, LPL, LZTS1, MIR320A, NPM2, NUDT18, PHYHIP, PIWIL2, POLR3D, PPP3CC, REEP4, SFTPC, SH2D4A, SLC18A1, SLC39A14, XPO7*
**8p21.2 - 8p21.1**	loss	19	*CHRNA2, CLU, EPHX2, MIR3622A, MIR3622B, PTK2B, SCARA3, STMN4, TRIM35*
**8p21.1**	loss	19	*C8orf80, CCDC25, ELP3, ESCO2, MIR4287, PBK, PNOC, SCARA5, ZNF395*
**8p22**	loss	18	*ASAH1, CNOT7, EFHA2, FGF20, FGL1, MIR383, MSR1, MTMR7, MTUS1, NAT1, NAT2, PCM1, PDGFRL, PSD3, SGCZ, SLC7A2, TUSC3, VPS37A, ZDHHC2*
**8p21.1 - 8p12**	loss	17	*DCTN6, DUSP4, EXTL3, FBXO16, FZD3, HMBOX1, INTS9, KIF13B, LEPROTL1, MBOAT4, MIR3148, MIR4288, TMEM66*
**8p21.3 - 8p21.2**	loss	17	*BIN3, C8orf58, CHMP7, EGR3, ENTPD4, KIAA1967, LOXL2, PDLIM2, PEBP4, R3HCC1, RHOBTB2, SLC25A37, SORBS3, TNFRSF10A, TNFRSF10B, TNFRSF10C, TNFRSF10D*
**8p23.3**	loss	17	*ARHGEF10, C8orf42, CLN8, DLGAP2, ERICH1, FBXO25, KBTBD11, MIR596, MYOM2, OR4F21, ZNF596*
**8p11.21**	loss	16	*AGPAT6, ANK1, AP3M2, C8orf40, CHRNA6, CHRNB3, DKK4, GINS4, GOLGA7, IKBKB, MIR486, MYST3, NKX6-3, PLAT POLB, SFRP1, SLC20A2, VDAC3, ZMAT4*
**8p11.22 - 8p11.21**	loss	16	*ADAM2, C8orf4, IDO1, IDO2*
**8p12**	loss	15	*C8orf41, DUSP26, FUT10, GSR, GTF2E2, MAK16, NRG1, PPP2CB, PURG, RBPMS, RNF122, TEX15, UBXN8, WRN*
**8p23.1 - 8p22**	loss	15	*C8orf48, DLC1, KIAA1456, LONRF1, MIR3926-1, MIR3926-2*
**8p11.23 - 8p11.22**	loss	14	*ADAM32, ADAM9, ASH2L, BAG4, C8orf86, DDHD2, EIF4EBP1, FGFR1, HTRA4, LETM2, LSM1, PLEKHA2, PPAPDC1B, STAR, TACC1, TM2D2, WHSC1L1*

Table S2. *Cont.*

**Cytoband region**	**CNV event**	**Number of tumors (n = 41)**	**Genes annotated to tumor-specific altered cytoband region**
**8p12 - 8p11.23**	loss	13	*KCNU1*
**8p23.2**	loss	13	*CSMD1*
**21q22.2 - 21q22.3**	loss	13	*BACE2, DSCAM, FAM3B, MIR3197, MX1, MX2, PCP4, PLAC4*
**21q22.3**	loss	13	*TMPRSS2*
**8p23.2 - 8p23.1**	loss	12	*AGPAT5, ANGPT2, MCPH1*
**8p11.23**	loss	12	*ADRB3, BRF2, ERLIN2, GOT1L1, GPR124, PROSC, RAB11FIP1, ZNF703*
**13q14.13**	loss	12	*C13orf18, CPB2, LCP1, ZC3H13*
**13q21.33**	loss	12	*DACH1,KLHL1*
**13q22.1**	loss	12	*KLF12, KLF5, PIBF1*
**16q24.1 - 16q24.2**	loss	12	*BANP, CA5A, COX4I1, COX4NB, FBXO31, FOXC2, FOXF1, FOXL1, IRF8, JPH3, KLHDC4, MAP1LC3B, MTHFSD, SLC7A5, ZCCHC14*
**16q24.2 - 16q24.3**	loss	12	*CTU2, CYBA, FAM38A, IL17C, MVD, RNF166, SNAI3, ZC3H18, ZFPM1, ZNF469*
**10q23.31**	loss	11	*ACTA2, ANKRD22, CH25H, FAS, IFIT1, IFIT1B, IFIT2, IFIT3, IFIT5, KIF20B, KLLN, LIPA, LIPF, LIPJ, LIPK, LIPM, LIPN, MIR107, PANK1, PTEN, RNLS, SLC16A12, STAMBPL1*
**16q23.2**	loss	11	*CDYL2, DYNLRB2*
**16q23.3 - 16q24.1**	loss	11	*ADAD2, ATP2C2, C16orf74, COTL1, CRISPLD2, FAM92B, GINS2, HSDL1, KCNG4, KIAA0182, KIAA0513, KIAA1609, KLHL36, LRRC50, MBTPS1, MIR1910, TAF1C, USP10, WFDC1, ZDHHC7*
**16q24.3**	loss	11	*ACSF3, ANKRD11, APRT, C16orf3, C16orf55, C16orf7, CBFA2T3, CDH15, CDK10, CDT1, CENPBD1, CHMP1A, CPNE7, DBNDD1, DEF8, DPEP1, FANCA, GALNS, GAS8, MC1R, PABPN1L, PRDM7, RPL13, SNORD68, SPATA2L, SPG7, SPIRE2, TCF25, TRAPPC2L, TUBB3, ZNF276, ZNF778*

Table S2. *Cont.*

Cytoband region	CNV event	Number of tumors (n = 41)	Genes annotated to tumor-specific altered cytoband region
**17p13.1**	loss	10	*ACADVL, ACAP1, ALOX12, ALOX12B, ALOX15B, ALOXE3, AMAC1L3, ARHGEF15, ASGR1, ASGR2, ATP1B2, AURKB, BCL6B, C17orf100, C17orf49, C17orf59, C17orf61, C17orf74, C17orf81, CCDC42, CD68, CHD3, CHRNB1, CLDN7, CLEC10A, CNTROB, CTC1, CTDNEP1, CYB5D1, DHRS7C, DLG4, DNAH2, DVL2, EFNB3, EIF4A1, EIF5A, FBXO39, FGF11, FXR2, GABARAP, GAS7, GLP2R, GPS2, GUCY2D, HES7, KCNAB3, KCTD11, KDM6B, KRBA2, LSMD1, MED31, MFSD6L, MIR195, MIR324, MIR3676, MIR4314, MIR497, MPDU1, MYH10, NDEL1, NEURL4, NLGN2, NTN1, ODF4, PER1, PFAS, PHF23, PIK3R5, PIK3R6, PLSCR3, POLR2A, RANGRF, RCVRN, RNASEK, RNF222, RPL26, SAT2, SCARNA21, SENP3, SHBG, SLC13A5, SLC16A11, SLC16A13, SLC25A35, SLC2A4, SNORA48, SNORA67, SNORD10, SOX15, SPDYE4, SPEM1, STX8, TEKT1, TMEM102, TMEM107, TMEM88, TMEM95, TNFSF12, TNFSF12-TNFSF13, TNFSF13, TNK1, TP53, TRAPPC1, TXNDC17, USP43, VAMP2, WDR16, WRAP53, XAF1, YBX2, ZBTB4*
**10q23.2 - 10q23.31**	loss	10	*ATAD1, PAPSS2*
**13q14.12 - 13q14.13**	loss	10	*COG3, FAM194B, GTF2F2, KCTD4, KIAA1704, NUFIP1, SIAH3, SLC25A30, SNORA31, SPERT, TPT1*
**13q14.13 - 13q14.2**	loss	10	*ESD, HTR2A, LRCH1*
**13q14.2**	loss	10	*ARL11, C13orf1, CAB39L, CDADC1, CYSLTR2, EBPL, FNDC3A, ITM2B, KCNRG, KPNA3, LPAR6, MED4, MIR3613, MLNR, NUDT15, PHF11, RB1, RCBTB1, RCBTB2, SETDB2, SUCLA2, TRIM13*
**13q21.33 - 13q22.1**	loss	10	*C13orf34, DIS3, MZT1*
**16q23.2 - 16q23.3**	loss	10	*ATMIN, BCMO1, C16orf46, C16orf61, CDH13, CENPN, CMIP, GAN, GCSH, HSBP1, HSD17B2, MIR3182, MLYCD, MPHOSPH6, NECAB2, OSGIN1, PKD1L2, PLCG2, SDR42E1, SLC38A8*
**6q14.3**	loss	10	*CGA, HTR1E, NT5E, SNORD50A, SNORD50B, SNX14, SYNCRIP, ZNF292*

Table S3. Complete table of investigated tumors showing clinical and pathological information. Where two scores are shown in the Gleason score (GS) column, the first is the GS for the upper cut-section of the tumor and the latter is the GS for the bottom cut-section of the tumor. MS = Muenster Bio-bank, RD = Rotterdam Bio-bank, L = tumor focus obtained from left side of the prostate, R = tumor focus obtained from the right side of the prostate, NA = not available. Matching blood specimens were analyzed from MS50, MS151, MS368, MS840, MS971, MS34, MS334, MS38 and normal prostate tissue from RD819. Samples from the left side of some unifocal prostate cancer cases: *MS34L, *MS173L and *MS334L had insufficient tumor quantity and were not analyzed. PSA, prostate-specific antigen.

Sample ID	Patient age at time of surgery (years)	Total Gleason score	Gleason score of individual focus	Pathological stage	Clinical stage	Prostate volume (cm^3)	Total tumor volume (cm^3)	Tumor volume of individual focus (cm^3)	Focality	Number of tumor foci in prostate	Pre-surgery PSA level (ng/mL)	PSA recurrence
MS50L<br>MS50R	66	4 + 3	3 + 4<br>4 + 3	pT3a	cT2c	20	2.1	0.7<br>1.4	multifocal	2	19.6	NA
MS151L<br>MS151R	61	4 + 3	4 + 3/3 + 3<br>4 + 3	pT3b	cT2c	19	2.85	0.76<br>2.09	multifocal	2	5.08	yes
MS183L<br>MS183R	59	4 + 3	3 + 4<br>3 + 4	pT3a	cT2b	35	7.35	0.35<br>7.0	multifocal	2	14.4	yes
MS210L<br>MS210R	62	3 + 3	3 + 3<br>3 + 3	pT3b	cT2c	32	8.64	4.16<br>4.48	multifocal	2	6.9	no
MS235L<br>MS235R	65	4 + 5	4 + 4/4 + 5<br>3 + 3/3 + 4	pT3a	cT2b	50	12.5	12.0<br>0.5	multifocal	2	3.68	yes
MS343L<br>MS343R	57	2 + 3	3 + 3<br>3 + 4/3 + 3	pT2b	cT2b	19	1.33	0.95<br>0.19	multifocal	3	6.34	no
MS368L<br>MS368R	65	4 + 5	3 + 4<br>4 + 5	pT3c	cT2b	32	13.76	2.56<br>11.2	multifocal	2	6.89	yes
MS407L<br>MS407R	51	3 + 4	3 + 4/4 + 3<br>3 + 3/3 + 4	pT2b	cT2c	27	1.35	0.81<br>0.54	multifocal	2	10.46	no

Table S3. *Cont.*

Sample ID	Patient age at time of surgery (years)	Total Gleason score	Gleason score of individual focus	Pathological stage	Clinical stage	Prostate volume (cm^3)	Total tumor volume (cm^3)	Tumor volume of individual focus (cm^3)	Focality	Number of tumor foci in prostate	Pre-surgery PSA level (ng/mL)	PSA recurrence
MS586L<br>MS586R	53	3 + 2	3 + 3<br>3 + 3	pT2c	cT1c	35	4.55	0.7<br>1.4	multifocal	5	10.63	no
MS840L<br>MS840R	66	4 + 3	3 + 4<br>3 + 4	pT3a	cT2c	24	2.64	1.44<br>0.48	multifocal	4	30.13	no
MS898L<br>MS898R	54	4 + 3	3 + 4/3 + 3<br>3 + 4	pT2c	cT1c	35	3.5	0.35<br>2.8	multifocal	3	6.48	no
MS946L<br>MS946R	61	3 + 2	3 + 3<br>3 + 3	pT3a	cT2b	45	6.75	6.3<br>0.45	multifocal	2	9.73	no
MS971L<br>MS971R	50	4 + 5	3 + 4<br>3 + 4	pT3a	cT2a	18	1.26	1.08<br>0.18	multifocal	2	14.78	no
RD819L<br>RD819R	52	NA	3 + 3<br>3 + 3	pT3a	cT1c	NA	NA	NA<br>NA	multifocal	2	6.5	no
*MS34L<br>MS34R	71	4 + 3	NA<br>4 + 3	pT2a	cT1c	38	4.56		unifocal	1	19.9	yes
MS38L<br>MS38R	62	3 + 4	3 + 3<br>3 + 3	pT3c	cT2c	33	22.4		unifocal	1	45.2	NA
MS78L<br>MS78R	66	3 + 3	3 + 3<br>3 + 3	pT3a	cT2b	44	3.08		unifocal	1	4.9	yes
MS99L<br>MS99R	57	3 + 4	3 + 3<br>3 + 3	pT3a	cT2c	34	4.42		unifocal	1	4.1	yes
*MS173L<br>MS173R	59	3 + 3	NA<br>3 + 3	pT3b	cT2b	24	1.2		unifocal	1	6.37	yes

Table S3. *Cont.*

Sample ID	Patient age at time of Surgery (years)	Total Gleason score	Gleason score of individual focus	Pathological stage	Clinical stage	Prostate volume (cm^3)	Total tumor volume (cm^3)	Focality	Number of tumor foci in prostate	Pre-Surgery PSA level (ng/mL)	PSA recurrence
*MS334L<br>MS334R	67	4 + 4	NA<br>3 + 3	pT3c	cT2c	28	15.96	unifocal	1	25.88	yes
MS470L<br>MS470R	64	3 + 4	3 + 3<br>3 + 3	pT3a	cT2b	49	11.27	unifocal	1	17.11	no
MS1096L<br>MS1096R	63	5 + 4	3 + 4/4 + 4<br>3 + 3	pT4	NA	75	45.75	unifocal	1	6.0	NA

© 2013 by the authors; licensee MDPI, Basel, Switzerland. This article is an open access article distributed under the terms and conditions of the Creative Commons Attribution license (http://creativecommons.org/licenses/by/3.0/).
